# Gel Cohesivity and Breast Augmentation: Applications to Clinical Practice

**DOI:** 10.1093/asjof/ojac088

**Published:** 2022-12-07

**Authors:** Michael C Edwards, Allen Gabriel, Jason Hammer, Hillary L Jewell, Mark L Jewell

**Affiliations:** Plastic surgeon in private practice, Las Vegas, NV, USA; Plastic surgeon in private practice, Vancouver, WA, USA; Executive medical director, Global Lead Plastics & Regenerative Medicine, Allergan Aesthetics, an AbbVie Company, Irvine, CA, USA; Board-certified nurse injector in private practice, Eugene, OR, USA; Plastic surgeon in private practice, Eugene, OR, USA

## Abstract

Matching breast implant gel cohesivity to the patient's needs based on anatomical and soft-tissue considerations and patient preferences is essential to optimizing aesthetic outcomes and patient satisfaction. Generally, gel cohesivity contributes to the feel and shape of the breast, which reflects volume distribution within the breast. Studies on the physical properties of silicone gel breast implants have established the relative cohesivity of implants from several manufacturers. Comprehensive patient education, tissue-based preoperative planning, and a thorough understanding of how implant properties may affect outcomes are recommended before breast augmentation. This review summarizes the literature on breast implant physical properties with a focus on gel cohesivity and translates gel cohesivity to practical considerations in choosing the right implant for the right patient in primary augmentation. Based on clinical experience, the authors generally recommend lower cohesivity implants for patients who desire a tapered upper pole or a natural appearance/“anatomic shape” as well as for those with a high BMI or a large amount of native breast tissue. Medium-cohesivity implants are most widely used and are appropriate for those with ptosis/augmentation mastopexy, patients with tight skin envelope, and for those seeking upper pole fullness. A high-cohesivity implant is recommended for patients desiring a round shape, patients with constricted lower poles, and patients with a postpartum loose envelope, a thin, soft tissue envelope, or fibrous breast tissue.

**Level of Evidence: 5:**

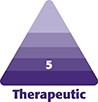

Many factors influence the choice of implant in primary breast augmentation, including patient preferences, breast shape, soft tissue characteristics, and implant properties, such as size, fill, profile, and gel cohesivity.^1‐3^ While all of these factors are important in deciding on an implant for a patient, matching the cohesivity of the implant to the needs of the patient based on anatomical and soft-tissue considerations and patient preferences is essential to optimizing aesthetic outcomes and patient satisfaction. In the context of silicone gel implants, cohesivity refers to the elasticity, or firmness (vs softness), of the gel.^4‐6^ The level of cohesivity affects the ability of the gel to retain its shape under force, including gravity, and can be assessed by standardized testing such as elastic response of the gel to maximally applied pressure.^4,6^ Under such testing, a less cohesive gel will show greater elastic deformation.

Gel cohesivity contributes to the feel of the implant (ie, softness vs firmness) as well as to the overall shape of the breast following augmentation. The fill volume of the implant determines the overall change in breast size, whereas shape reflects volume distribution of the implant within the breast; implants with more cohesive gels are less likely to spread within the breast pocket compared with less cohesive gels.^1,2,6^ Gel cohesivity is therefore important to optimizing volume distribution in order to achieve the desired breast shape. Other factors contributing to volume distribution include the patient's anatomy and the shape and placement of the implant.^6,7^ Greater gel cohesivity, a higher gel-shell fill ratio, and increased adherence of the gel to the shell tend to enhance maintenance of implant shape.^7^

Not only is the concept of gel cohesivity important to the plastic surgeon, it is also important for patients to understand the concept. Patients are increasingly being encouraged to participate in the choice of implant selection under the guidance of the aesthetic team^2,8,9^ and would benefit from education during the consultation process on factors, including cohesivity, that will impact their aesthetic outcomes. The goal of this manuscript is to provide guidance to plastic surgeons on the role of gel cohesivity in primary breast augmentation by examining the physical properties of silicone breast implants and applying this information to clinical practice. The role of patients in the decision-making process is also discussed.

## COHESIVITY OF SILICONE GEL BREAST IMPLANTS: GEL FORMATION AND PHYSICAL PROPERTIES

The gel in silicone breast implants is composed of silicone polymers called polydimethylsiloxanes (PDMS).^10^ Cross-linking of the polymer chains is achieved by substituting the methyl groups on the PDMS polymer chain with ethenyl and hydride groups, which interact during a hydrosilylation reaction to form a 2-carbon bridge between polymers.^10,11^ The degree of silicone cross-linking determines the level of cohesivity, with a higher number of cross-linking sites associated with greater cohesivity.^10,12,13^

Although there is no established standard for measuring silicone gel cohesivity,^6^ 2 studies employing similar techniques have yielded consistent results.^4,6^ Both studies evaluated breast implant cohesivity (elastic deformation) using a BTC-2000 system (SRLI Technologies, Franklin, TN), which applies negative pressure on the gel from an implant with the shell removed while measuring material deformation over time using a synchronized target laser. Gel cohesivity was measured as distance in millimeters obtained up to the point of maximum negative pressure applied (ie, 15 mm Hg), with higher values reflecting a less cohesive gel.

In the first study, the cohesivity of a range of round and shaped silicone breast implants from Sientra (Santa Barbara, CA), Mentor (Santa Barbara, CA), and Allergan (Irvine, CA) was assessed and compared.^4^ Among the anatomically shaped implants tested, the Natrelle 410 implant containing TruForm 3 gel (Allergan, Irvine, CA) had the highest cohesivity, and the Sientra HSC-Plus implant had the lowest cohesivity, with statistically significant differences observed for the Sientra implant compared with the Allergan and Mentor implants. Although the Natrelle 410 implant is no longer sold, the current Natrelle Inspira implants offer the option of TruForm 3 gel. Of the smooth, round implants tested, the Sientra device with HSC gel was the most cohesive, and the Allergan device with responsive gel (TruForm 1) was the least cohesive; differences between all devices were statistically significant.

In the second study, gel cohesivity, resistance to gel deformation, and energy absorption (softness) of round and shaped silicone breast implants manufactured by Sientra, Mentor, Allergan, and Establishment Labs (Alajuela, CR) were measured.^6^ Among the shaped implants tested, an implant containing TruForm 3 had significantly greater gel cohesivity than Mentor MemoryShape and Sientra Textured Shaped implants (*P* < .0001). Of round implants tested, the rank order from highest to lowest gel cohesivity was Natrelle Inspira Cohesive (TruForm 3), Sientra Luxe (HSC-Plus), Motiva Round (ProgressiveGel Plus), Natrelle Inspira SoftTouch (TruForm 2), MemoryGel (Cohesive II), Sientra Round (HSC), MemoryGel (Cohesive I), and Natrelle Inspira Responsive (TruForm 1). Resistance to gel deformation (stiffness) using the BTC-2000 system was also measured in this study, with rank results comparable with those observed with cohesivity testing.

In a separate study, resistance to gel deformation (stiffness) was measured in shaped implants using a tractiometer (Lloyd Instruments, Bognor Regis, UK) with the implant shell still on.^14^ The system was programed to penetrate a probe on the implant from a distance of 20 mm from the maximum projection point, and the reaction to compression was measured in Newtons; higher resistance indicates greater stiffness. Stiffness was comparable for the Natrelle 410 with TruForm 3, Mentor MemoryShape Contour Profile Gel implant with Cohesive III, and Sebbin Naturgel implants (Paris, France) but was lower with the Natrelle 410 with TruForm 2. While the rank order of stiffness of implants containing TruForm 2, TruForm 3, and Cohesive III gels mirrors that described above for cohesivity, implant stiffness depends not only on cohesivity but also on other aspects, such as shell characteristics and the degree of filling.

**Figure 1.**
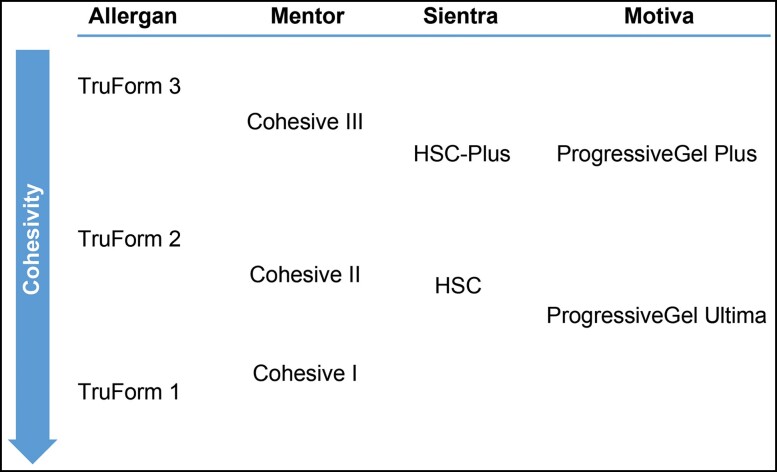
Relative cohesivity levels of different silicone gels.^4,6^ HSC, high strength cohesive.

Figure 1 presents a schematic of the relative cohesivity levels of different breast implant gel types based on studies of physical properties.^4,6^ Manufacturers offer implant gels across a range of cohesivity.

## TRANSLATING PHYSICAL PROPERTIES OF IMPLANTS TO THE CLINICAL DECISIONS FOR PATIENTS WITHIN A SURGICAL PRACTICE

Understanding the impact of cohesivity on clinical outcomes allows for a critical assessment of the potential clinical performance characteristics of a particular implant.^3^ With increasing cohesivity, there is generally less rippling/wrinkling and greater maintenance of upper pole dimension and projection, but also less softness.^3,5,6,15,16^ In addition, more cohesive implants provide the ability to shape the breast as opposed to just adding volume.^5,6^ Optimizing volume distribution (ie, lower vs upper pole volume) is one aesthetic goal of breast augmentation therapy. While patient preferences may differ, an upper pole/lower pole distribution of 45:55 is considered aesthetically desirable by current US beauty standards.^17^ Compared with less cohesive implants, more cohesive implants produce a fuller upper pole in the upright position, with more even gel distribution throughout the implant, and are thus less likely to result in a centrally full appearance and a collapsed upper pole.^5^ The less cohesive a gel, the greater the tendency to redistribute into the lower portion of the implant or the breast pocket.^6^ However, highly cohesive implants pose a greater risk of anterior-posterior flipping, particularly with higher projection and larger volume implants.^3,18^ With form-stable implants that have a rounded anterior profile and a flat posterior profile, anterior-posterior flipping may result in a visible breast deformity with a flattened appearance of the implant and palpable/visible implant borders.

The benefit of a shaped vs a round implant with regard to volume distribution is negated if the shaped implant is unable to retain its form (ie, form stability) when moved from a horizontal to a vertical position secondary to decreased cohesivity.^6^ Under certain circumstances, high-cohesivity round implants may produce results indistinguishable from those achieved with a shaped implant.^19,20^ However, this may not be the case in patients with thin soft tissue and concave upper poles^19^ or with the latest high-cohesivity round and shaped implants.^21^

While speculative, it has been suggested that implants with a higher fill-to-shell ratio and more cohesive gels are less likely to rupture due to fewer folds resulting from movement of the gel within the implant shell.^22^ Should a rupture occur, the gel is less likely to show significant migration compared with less cohesive gels manufactured before 1992,^23^ although the higher cohesivity gels introduced with third-generation implants compared with previous generations of implants have a very low risk of gel migration.^24^

## CHOOSING AN IMPLANT

Comprehensive patient education, understanding of the patient's anatomy and tissue characteristics, and a thorough appreciation of how implant properties may affect outcomes are recommended prior to breast augmentation.^8,25^ In recent years, the practice of breast augmentation has shifted toward a more collaborative process taking into account the patient's wishes and involving the patient as a partner in implant selection.^2,8,9^ The needs of the patient, including the desired appearance, can be ascertained upon consultation, which in some practices may be facilitated by a nurse practitioner, patient coordinator, or other office staff.^26^ Patient education can take the form of face-to-face counseling, patient sizing tools, and consultation tools such as TouchMD (Cedar City, UT), and 3-dimensional computer modeling systems, such as Vectra X3 (Canfield Scientific, Parsippany, NJ) and Crisalix VR (Crisalix Virtual Aesthetics, Lausanne, Switzerland).^3,9,27‐29^ Three-dimensional breast imaging permits surgeons to model the influence of implant parameters on breast shape and facilitates communication with patients about the expected breast shape and size with different implants, which may help in setting patient expectations.^27,29^

Patients should be involved in device selection, including being educated on the available devices and the importance of tissue-based planning and optimal implant selection for their tissues.^2,8^ Implant–soft-tissue dynamics impact the short-term and long-term results of breast augmentation.^1,30^ While pressure from the breast implant on overlying soft tissues contributes to breast shape, the overlying soft tissues also exert biomechanical stress on the implant. This counter-pressure depends on multiple factors, such as the compliance of the envelope, the layers of parenchyma and/or muscle overlying the implant and the envelope, and the projection and cohesivity of the implant. The interaction of implant and tissue forces over time determines long-term aesthetic outcomes; for these reasons, evaluation of the patient's tissues should be taken into consideration when setting realistic goals and recommending a specific implant or gel cohesivity and appropriate surgical techniques.^31^ Other tissue factors to consider include the amount of native breast tissue, parity, and degree of athleticism.^8,32^ In addition to evaluating a patient's tissues, key measurements should be taken, including upper-pole pinch, breast base width, degree of ptosis, sternal notch-to-nipple distance, and nipple-to-inframammary fold distance.^3,30^ The collaboration between surgeon and patient in selecting an implant is dependent on a complete assessment of all factors that might influence implant choice.

Decision-support processes have been developed to facilitate preoperative decisions in breast augmentation with respect to implant–soft-tissue dynamics. The High Five system for implant selection and preoperative planning prioritizes decisions related to optimal soft-tissue coverage/pocket location; implant volume; implant type, size, and dimensions; inframammary fold position; and incision location.^33^ Subsequent modifications of the High Five system have aimed to update and simplify the process.^8,34^ Increased risk for implant malposition based on physical characteristics, implant choice, and planned lowering of the inframammary fold should be assessed preoperatively and appropriate patients considered for inframammary fold anchoring by suturing.^35^

An understanding of the elements of cohesivity and associated differences between implants will enable patients to make better informed decisions regarding implant selection in order to achieve their goals and will also help establish realistic expectations regarding short-term and long-term outcomes of breast augmentation. Ultimately, setting realistic expectations for clinical outcomes will enhance patient satisfaction. Several clinical trials have documented high rates of satisfaction after breast augmentation that are in the 19th percentile,^36‐40^ but individual satisfaction is important in practice and may reduce the likelihood of reoperation due to unmet sizing expectations or undesired shape. A retrospective review of 494 patients who underwent breast augmentation at a single surgeon's practice, implementing an approach that included extensive preoperative patient education and tissue-based planning with 3D imaging, reported high levels of patients satisfaction and low rates of reoperation for any reason (2.7%) or due to dissatisfaction with size (0.6%).^34^ One author has gone 11.5 years without a reoperation due to incorrect size selection.^41^

## DECISION TREE

**Figure 2.**
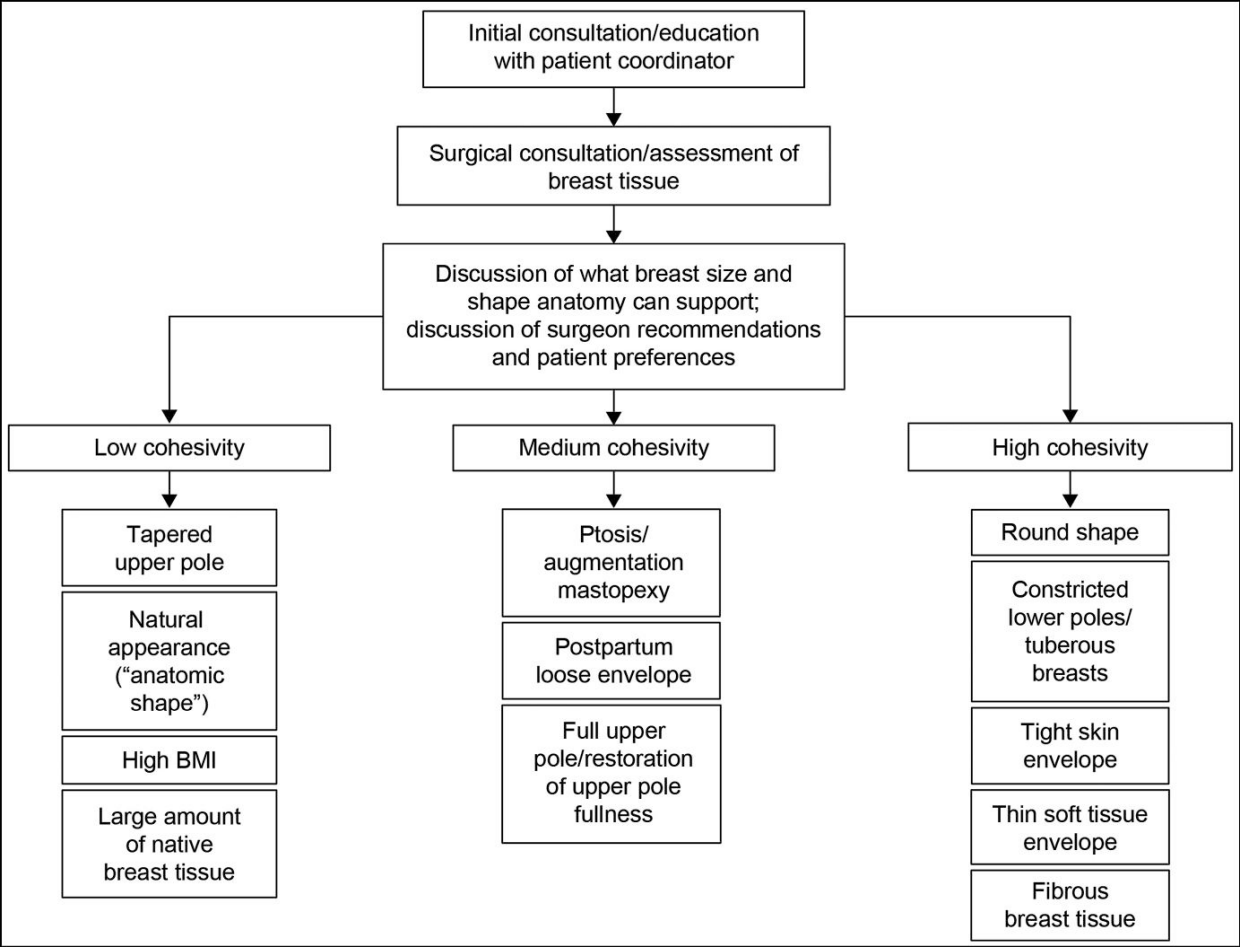
Practical considerations in matching patients with gel implant cohesivities.

Practical considerations for choosing the right implant for the right patient with a focus on gel cohesivity and surgical goals based on authors’ clinical experience are shown in Figure 2. The guidelines match the cohesivity of the gel with patient desires and anatomical considerations (“tissues and wishes”) and begin with an initial consultation and education with a patient coordinator, who may be a nurse practitioner. As described above, the next step involves surgical consultation and assessment of breast tissue. This step is followed by a discussion of what the patient's breast size and shape anatomy can support in relation to the surgeon recommendations and patient preferences. The branch point in distinguishing the needs of different patients is to decide which level of gel cohesivity will support desired outcomes in the context of patient anatomy (eg, more cohesive gels can be used to shape the breast and less cohesive gels can be used to restore volume). A good outcome can be achieved depending on how the gel is selected and the plane of placement. Subfascial placement in the appropriate patients (eg, those with adequate tissue to cover the implant) has benefits such as lower reoperation rates compared with submuscular placement, lower incidences of capsular contracture compared with subglandular placement, and lower incidences of ripples, infection, and animation deformity compared with any other plane of placement.^42^ If a patient's expectations for size and shape are unrealistic based on physical measurements, the consultation should be stopped rather than proceeding with an ill-advised surgery (eg, using an implant that is too wide or too large as a workaround to ptosis) that will lead to a dissatisfied patient. In the case studies that follow, written consent was provided, by which the patients agreed to the use and analysis of their data.

### Lower Cohesivity Gel Implants

Dual-plane augmentation with a lower cohesivity gel can produce a tapered upper breast for patients who desire a more natural appearance/“anatomic shape.” Those with high BM) would likely benefit from greater breast projection achievable with a round, less cohesive implant, because the greater gel volume in the lower pole upon standing would make the implant project forward.^5^ In addition, patients with large amounts of native breast tissue tend to be good candidates for round, less cohesive implants. Less cohesive implants may also be appropriate for patients who desire a more natural appearance of the upper breast.^2^

#### Case Illustration

**Figure 3.**
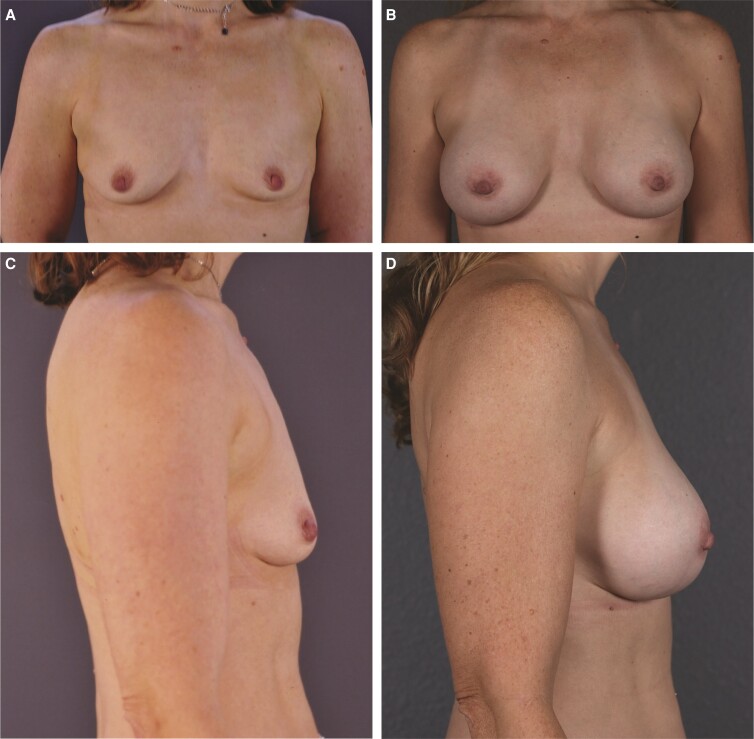
Example of outcomes with a lower cohesivity gel implant in a 38-year-old female with Natrelle Inspira responsive (TruForm 1) smooth, round, full profile 345-cc implant (Allergan, Irvine, CA). Frontal view: (A) preimplantation and (B) 3 years postimplantation; lateral view: (C) preimplantation and (D) 6 months postimplantation.

The patient was a healthy 35-year-old female, measuring 5′3″ (160 cm) and weighing 105 lbs (47.6 kg). She had 3 previous pregnancies and possible plans for additional childbearing. The patient was very athletic and physically active (ie, weightlifting, swimming, biking, and running). During initial consultation, she indicated that she would like to increase her preoperative A-cup to a C-cup through bilateral augmentation and expressed a desire for a natural, tapered upper breast with accentuated upper breast fullness. On examination, the patient was found to have type II breasts, with very little breast tissue, moderate constriction, and a tight skin envelope in the lower part of the breast (Figure 3A, C). Chest wall circumference was 28″. The nipples were 17.5 cm from the sternal notch bilaterally, nipple-to-nipple distance across was 18 cm, and nipple-to-fold distance was 6 cm. Base diameter was 11.5 cm. The patient had 32 mm of upper-pole pinch. During the consultation, biplanar augmentation was discussed.

Given the small amount of native breast tissue and the desire for a natural appearing upper pole, a responsive gel implant (TruForm 1) was selected. Allergan Natrelle Inspira smooth, round, full profile (smooth surface, responsive gel [SRF]) 295-cc implant was used, along with lowering of the fold to produce an aesthetically pleasing clinical outcome. The implant was inserted using biplanar technique through an incision in the inframammary fold, and nipple telescoping was also performed (Figure 3B, D).

### Medium Cohesivity Gel Implants

A medium-cohesivity implant tends to be a suitable choice for most patients seeking breast augmentation and is appropriate for patients who desire a fuller upper pole. Restoration of upper breast roundness can also be achieved with medium cohesivity in postpartum patients. A medium cohesivity implant can provide extra firmness in women with deficiency due to ptosis. For a female who is postpartum and has a loose envelope, a medium cohesive gel in the subfascial location would allow greater restoration of upper breast fullness.

#### Case Illustration

**Figure 4.**
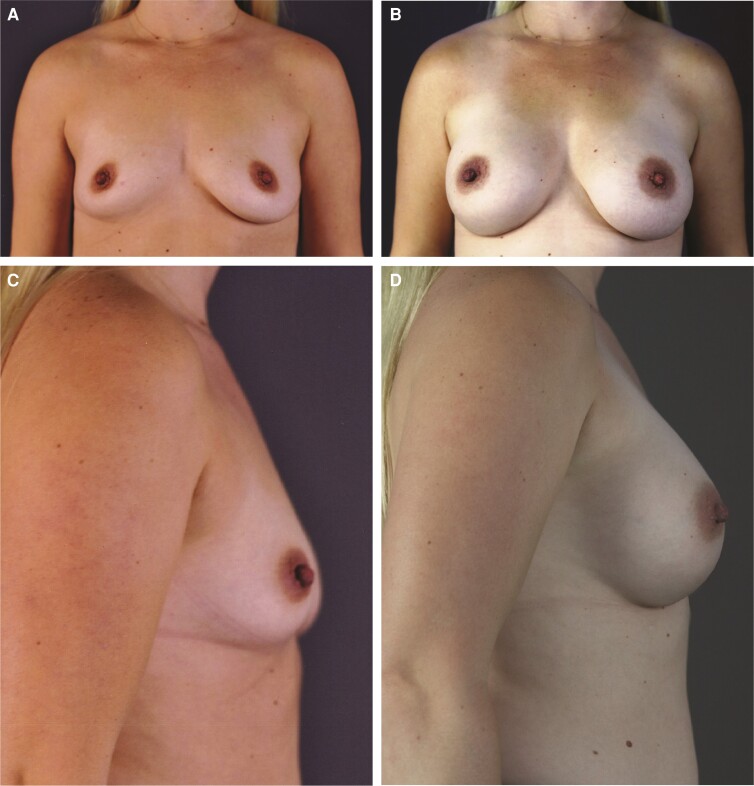
Example of outcomes with a medium-cohesivity gel implant in a 31-year-old female patient with Natrelle Inspira smooth, round soft touch (TruForm 2) full profile (SSF) 365-cc implant on the right and Natrelle Inspira SSF 325-cc implant (Allergan, Irvine, CA) on the left. Frontal view: (A) preimplantation and (B) 9 months postimplantation. Lateral view: (C) preimplantation and (D) 6 months postimplantation. SSF, smooth surface, soft touch gel.

The patient was a 31-year-old female, measuring 5′4″ (162.6 cm) and weighing 145 lbs (65.8 kg). She had 2 previous pregnancies and was active in Cross-Fit gym activities. During initial consultation, the patient said that she desired more symmetric breasts and wanted to increase her preoperative A/B-cup size to a C/D-cup size. Examination revealed some side-to-side asymmetry, with the left breast more ptotic than the right (Figure 4A, C). The right nipple was at 20 cm from the sternal notch, and the left nipple was at 20.5 cm from the sternal notch. Nipple-to-nipple distance across was 19 cm. Nipple-to-fold distance was 8 cm on the right and 9 cm on the left. The right fold appeared to be lower than the left. Chest wall circumference was 32″. The patient had 44 mm of upper-pole pinch.

During the consultation, the plastic surgeon and the patient decided on an asymmetric breast augmentation with a larger Allergan Natrelle Inspira smooth, round soft touch (TruForm 2) full profile (SSF) 365-cc implant on the right and an Allergan Natrelle Inspira SSF 325-cc implant on the left, using retromammary-subfascial placement through an incision in the inframammary fold. Clinical outcomes were aesthetically pleasing, and the patient was satisfied with the results (Figure 4B, D). She did not have animation deformity due to the retromammary-subfascial implant placement.

### High Cohesivity Gel Implants

Round high-cohesivity implants are useful for providing a definitive round shape if that is the patient preference. In patients with constricted lower poles/tuberous breasts, either a shaped or a more cohesive round implant allows for expansion of the lower pole.^43^ A high-cohesivity implant works similarly to a tissue expander in patients with tuberous breasts or a tight skin envelope to stretch the lower pole into a rounder breast shape.^44^ Patients with a thin soft tissue envelope, who tend to have lower BMIs, are generally candidates for more cohesive breast implants.^2,5^ Additionally, high-cohesivity implants should be considered for patients with more fibrous breast tissue that resists stretch.

#### Case Illustration

**Figure 5.**
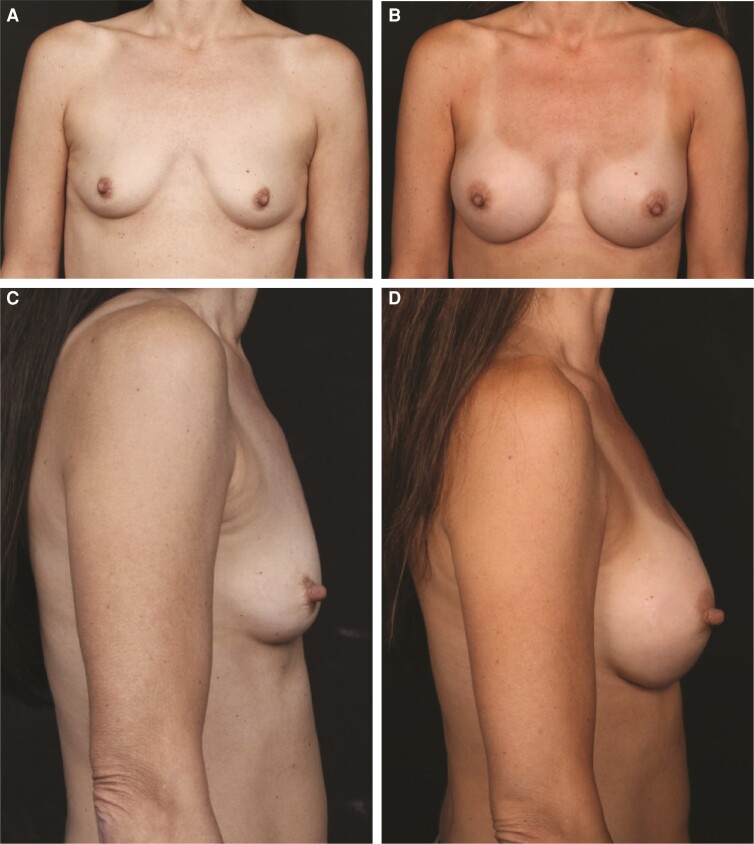
Example of outcomes with a high-cohesivity gel implant in a 52-year-old female patient with Natrelle Inspira cohesive (TruForm 3) smooth, round moderate profile 310-cc implant (Allergan, Irvine, CA). Frontal view: (A) preimplantation and (B) 2.5 years postimplantation. Lateral view: (C) preimplantation and (D) 6 months postimplantation.

The patient was a healthy 52-year-old female, measuring 5′7″ (170 cm) and weighing 133 lbs (60.3 kg). She had 1 previous pregnancy and no plans for additional childbearing. The patient was very athletic and physically active (ie, weightlifting and running). During initial consultation, she indicated that she would like to increase her preoperative A-cup through bilateral augmentation and expressed desire for a natural, tapered upper breast but fuller look. The patient always struggled with achieving her desired amount of cleavage and has not been able to do so with a bra. Her main goal was to attain a natural look and enhanced cleavage to improve her confidence. On examination, the patient was found to have type II breasts, with very little breast tissue, mild constriction, and a tight skin envelope in the lower part of the breast (Figure 5A, C). The nipples were 20.0 cm from the sternal notch on the left and 19.5 on the right, and nipple-to-fold distance was 6 cm on the left and 6.5 cm on the right. Base diameter was 13.0 cm. The patient had 2 cm of upper-pole pinch.

The patient's small amount of native breast tissue and insufficient pinch thickness made her a candidate for biplanar augmentation to achieve her desired natural appearance. Given the nature of cohesive gels and their known stability, the decision was made collaboratively with the patient to move forward with cohesive implants. After external sizing and use of 3D imaging as a discussion tool, the implant was selected.

The tradeoffs of subglandular/subfascial and biplanar augmentation were discussed with the patient given her level of activity, and different projections were considered because the patient was a candidate for both smooth, round, low plus profile (smooth surface, cohesive gel [SCLP]) and smooth, round moderate profile (smooth surface, cohesive gel [SCM]) implant styles. Given the plane of placement and the patient's desire for cleavage stability, a moderate-profile implant was recommended. It was explained to the patient that once a certain size (ie, 300 cc) is reached in a low-profile implant, some projection is required with cohesive implants because they do not collapse compared with responsive gels (eg, TruForm 1). For an aesthetically pleasing breast shape, projection is also needed, and an SCLP implant would result in the breasts looking flat and wide with no real shape. Therefore, an Allergan Natrelle Inspira cohesive (TruForm 3) SCM 310-cc implant was selected, along with mild lowering of the fold to produce the desired clinical outcome. The implant was inserted using biplanar technique through an incision in the inframammary fold measuring 5 cm (Figure 5B, D).

## CONCLUSIONS

The availability of silicone breast implants across a range of cohesivity levels allows surgeons to match the gel cohesivity to the patient's needs and portends a better aesthetic outcome and a satisfied patient. Selection of the cohesivity level of an implant should ideally be made by the patient in partnership with the surgeon, considering patient preferences and goals as well as anatomical and soft-tissue considerations. While a medium cohesivity implant may serve the needs of the majority of patients seeking augmentation, less cohesive implants may be preferred by patients desiring a softer feel or lower pole fullness; conversely, more cohesive implants may be preferable in patients with a thin, soft tissue envelope and/or a low BMI. Future studies that evaluate the effects of different cohesivity levels on long-term outcomes of breast augmentation, such as ptosis development, would be instructive.

## References

[1] Tebbetts JB . A system for breast implant selection based on patient tissue characteristics and implant-soft tissue dynamics. Plast Reconstr Surg. 2002;109(4):1396–1409. doi: 10.1097/00006534-200204010-00030 11964998

[2] Kortesis BG , Bharti G. Maximizing aesthetics and patient selection utilizing Natrelle Inspira line implants in aesthetic breast surgery. Plast Reconstr Surg. 2019;144(1):30s–36s. doi: 10.1097/prs.0000000000005960 31246758

[3] Warren Peled A , Disa JJ. Outcomes utilizing Inspira implants in primary aesthetic and reconstructive surgery. Plast Reconstr Surg. 2019;144(1):60s–65s. doi: 10.1097/prs.0000000000005951 31246762 PMC8257083

[4] Kinney BM , Jeffers LL, Ratliff GE, Carlisle DA. Silicone gel breast implants: science and testing. Plast Reconstr Surg. 2014;134(1 Suppl):47S–56S. doi: 10.1097/prs.0000000000000349 25057749

[5] Gabriel A , Maxwell GP. The science of cohesivity and elements of form stability. Plast Reconstr Surg. 2019;144(1):7s–12s. doi: 10.1097/prs.0000000000005959 31246755

[6] Jewell ML , Bengtson BP, Smither K, et al Physical properties of silicone gel breast implants. Aesthet Surg J. 2019;39(3):264–275. doi: 10.1093/asj/sjy103 29718087 PMC6376345

[7] Maxwell GP , Gabriel A. Breast implant design. Gland Surg. 2017;6(2):148–153. doi: 10.21037/gs.2016.11.09 28497018 PMC5409902

[8] Adams WP Jr , Small KH. The process of breast augmentation with special focus on patient education, patient selection and implant selection. Clin Plast Surg. 2015;42(4):413–426. doi: 10.1016/j.cps.2015.06.001 26408433

[9] Ubbink DT , Santema TB, Lapid O. Shared decision-making in cosmetic medicine and aesthetic surgery. Aesthet Surg J. 2016;36(1):NP14–NP19. doi: 10.1093/asj/sjv107 26104476

[10] Calobrace MB , Capizzi PJ. The biology and evolution of cohesive gel and shaped implants. Plast Reconstr Surg. 2014;134(1 Suppl):6S–11S. doi: 10.1097/prs.0000000000000347 25057753

[11] Brook MA . The chemistry and physical properties of biomedical silicones. In: Peters W, Brandon H, Jerina KL, Wolf C, Young VL, eds. Biomaterials in Plastic Surgery. Woodhead Publishing; 2012:52–67.

[12] Kappel RM , Klunder AJH, Pruijn GJM. Silicon chemistry and silicone breast implants. Eur J Plast Surg. 2014;37(3):123–128. doi: 10.1007/s00238-013-0914-4

[13] Gabriel A , Maxwell GP. The evolution of breast implants. Clin Plast Surg. 2015;42(4):399–404. doi: 10.1016/j.cps.2015.06.015 26408431

[14] Atlan M , Bigerelle M, Larreta-Garde V, et al Characterization of breast implant surfaces, shapes, and biomechanics: a comparison of high cohesive anatomically shaped textured silicone, breast implants from three different manufacturers. Aesthetic Plast Surg. 2016;40(1):89–97. doi: 10.1007/s00266-015-0603-8 26746882

[15] Panettiere P , Marchetti L, Accorsi D. Soft cohesive silicone gel breast prostheses: a comparative prospective study of aesthetic results versus lower cohesivity silicone gel prostheses. J Plast Reconstr Aesthet Surg. 2007;60(5):482–489. doi: 10.1016/j.bjps.2006.04.020 17399656

[16] Abramo AC , Scartozzoni M, Lucena TW, Sgarbi RG. High- and extra-high-profile round implants in breast augmentation: guidelines to prevent rippling and implant edge visibility. Aesthetic Plast Surg. 2019;43(2):305–312. doi: 10.1007/s00266-018-1264-1 30483933

[17] Mallucci P , Branford OA. Concepts in aesthetic breast dimensions: analysis of the ideal breast. J Plast Reconstr Aesthet Surg. 2012;65(1):8–16. doi: 10.1016/j.bjps.2011.08.006 21868295

[18] Jong J , Gabriel A, Trekell M, et al Cohesive round implants and the risk of implant flipping. Plast Reconstr Surg Glob Open. 2020;8(12):e3321. doi: 10.1097/gox.0000000000003321 33425624 PMC7787287

[19] Friedman T , Davidovitch N, Scheflan M. Comparative double blind clinical study on round versus shaped cohesive gel implants. Aesthet Surg J. 2006;26(5):530–536. doi: 10.1016/j.asj.2006.08.004 19338941

[20] Rubi CG , Lozano JA, Pérez-Espadero A, Leache ME. Comparing round and anatomically shaped implants in augmentation mammaplasty: the experts’ ability to differentiate the type of implant. Plast Reconstr Surg. 2017;139(1):60–64. doi: 10.1097/prs.0000000000002896 28027228

[21] Jewell ML . Comparing round and anatomically shaped implants in augmentation mammaplasty: the experts’ ability to differentiate the type of implant. Plast Reconstr Surg. 2017;140(4):626e–627e. doi: 10.1097/prs.0000000000003734 28632642

[22] Brandon HJ , Taylor ML, Powell TE, Walker PS. Morphology of breast implant fold flaw failure. J Long Term Eff Med Implants. 2006;16(6):441–450. doi: 10.1615/jlongtermeffmedimplants.v16.i6.40 17956211

[23] Hillard C , Fowler JD, Barta R, Cunningham B. Silicone breast implant rupture: a review. Gland Surg. 2017;6(2):163–168. doi: 10.21037/gs.2016.09.12 28497020 PMC5409893

[24] Zambacos GJ , Molnar C, Mandrekas AD. Silicone lymphadenopathy after breast augmentation: case reports, review of the literature, and current thoughts. Aesthetic Plast Surg. 2013;37(2):278–289. doi: 10.1007/s00266-012-0025-9 23354761

[25] Adams WP J . The process of breast augmentation: four sequential steps for optimizing outcomes for patients. Plast Reconstr Surg. 2008;122(6):1892–1900. doi: 10.1097/PRS.0b013e31818d20ec 19050543

[26] Tebbetts JB , Tebbetts TB. An approach that integrates patient education and informed consent in breast augmentation. Plast Reconstr Surg. 2002;110(3):971–978. doi: 10.1097/00006534-200209010-00039. discussion 979-981. 12172168

[27] Roostaeian J , Adams WP Jr. Three-dimensional imaging for breast augmentation: is this technology providing accurate simulations? Aesthet Surg J. 2014;34(6):857–875. doi: 10.1177/1090820x14538805 24970274

[28] Epstein MD , Scheflan M. Three-dimensional imaging and simulation in breast augmentation: what is the current state of the art? Clin Plast Surg. 2015;42(4):437–450. doi: 10.1016/j.cps.2015.06.013 26408435

[29] de Runz A , Boccara D, Bertheuil N, et al Three-dimensional imaging, an important factor of decision in breast augmentation. Ann Chir Plast Esthet. 2018;63(2):134–139. doi: 10.1016/j.anplas.2017.07.019 28911890

[30] Tebbetts JB . Dual plane breast augmentation: optimizing implant-soft-tissue relationships in a wide range of breast types. Plast Reconstr Surg. 2001;107(5):1255–1272. doi: 10.1097/00006534-200104150-00027 11373572

[31] Vegas MR , Martin del Yerro JL. . Stiffness, compliance, resilience, and creep deformation: understanding implant-soft tissue dynamics in the augmented breast: fundamentals based on materials science. Aesthetic Plast Surg. 2013;37(5):922–930. doi: 10.1007/s00266-013-0197-y 23943051

[32] Hedén P , Brown MH, Luan J, et al Delphi study consensus recommendations: patient selection and preoperative planning measurements for Natrelle 410. Plast Reconstr Surg Glob Open. 2015;3(11):e556. doi: 10.1097/gox.0000000000000510 26893981 PMC4727708

[33] Tebbetts JB , Adams WP. Five critical decisions in breast augmentation using five measurements in 5 minutes: the high five decision support process. Plast Reconstr Surg. 2005;116(7):2005–2016. 16327616

[34] Diaz JF . Review of 494 consecutive breast augmentation patients: system to improve patient outcomes and satisfaction. Plast Reconstr Surg Glob Open. 2017;5(10):e1526. doi: 10.1097/gox.0000000000001526 29184739 PMC5682175

[35] Campbell CF , Small KH, Adams WP Jr. The inframammary fold (IMF) fixation suture: proactive control of the IMF in primary breast augmentation. Aesthet Surg J. 2016;36(5):619–623. doi: 10.1093/asj/sjv178 26399314

[36] Cunningham B . The mentor core study on silicone MemoryGel breast implants. Plast Reconstr Surg. 2007;120(7 Suppl 1):19S–29S. doi: 10.1097/01.prs.0000286574.88752.04 18090810

[37] Hammond DC , Migliori MM, Caplin DA, et al Mentor contour profile gel implants: clinical outcomes at 6 years. Plast Reconstr Surg. 2012;129(6):1381–1391. doi: 10.1097/PRS.0b013e31824ecbf0 22327894

[38] Spear SL , Murphy DK. Natrelle round silicone breast implants: core study results at 10 years. Plast Reconstr Surg. 2014;133(6):1354–1361. doi: 10.1097/PRS.0000000000000021 24867717 PMC4819531

[39] Maxwell GP , Van Natta BW, Bengtson BP, Murphy DK. Ten-year results from the Natrelle® 410 anatomical form-stable silicone breast implant core study. Aesthet Surg J. 2015;35(2):145–155. doi: 10.1093/asj/sju084 25717116 PMC4399443

[40] Stevens WG , Calobrace MB, Alizadeh K, et al Ten-year core study data for Sientra’s Food and Drug Administration-approved round and shaped breast implants with cohesive silicone gel. Plast Reconstr Surg. 2018;141(4S Sientra Shaped and Round Cohesive Gel Implants):7s–19s. doi: 10.1097/prs.0000000000004350 29595714

[41] Jewell ML . Silicone gel breast implants at 50: the state of the science. Aesthet Surg J. 2012;32(8):1031–1034. doi: 10.1177/1090820X12461649 23012658

[42] Brown T . A comprehensive outcome review of subfascial breast augmentation over a 10-year period. Plast Reconstr Surg. 2020;146(6):1249–1257. doi: 10.1097/prs.0000000000007333 33234953

[43] Mallucci PL . 10-year experience using inspira implants: a review with personal anecdote. Plast Reconstr Surg. 2019;144(1S Utilizing a Spectrum of Cohesive Implants in Aesthetic and Reconstructive Breast Surgery):37s–42s. doi: 10.1097/prs.0000000000005948 31246759

[44] Avvedimento S , Montemurro P, Cigna E, et al Quantitative analysis of nipple to inframammary fold distance variation in tuberous breast augmentation: is there a progressive lower pole expansion? Aesthetic Plast Surg. 2021;45(5):2017–2024. doi: 10.1007/s00266-021-02363-8 34100102

